# Two years of COVID-19: many lessons, but will we learn?

**DOI:** 10.2807/1560-7917.ES.2022.27.10.2200222

**Published:** 2022-03-10

**Authors:** David L. Heymann, Helena Legido-Quigley

**Affiliations:** 1 London School of Hygiene and Tropical Medicine, London, United Kingdom; 2 Saw Swee Hock School of Public Health, National University of Singapore, Singapore

It has been two years since 11 March 2020 when the Director General of the World Health Organization announced that coronavirus disease (COVID-19) had become pandemic [1]. Since then, the disease has had devastating consequences worldwide with more than 6 million reported deaths by early March 2022, and an estimated economic damage of around 84.54 trillion USD to the world’s economy in 2020 [2,3]. In the European Union, COVID-19 has resulted in an unprecedented economic contraction with real gross national product falling by 6.1% in 2020.

Two years into the pandemic, there are many lessons to be learned as resurgent transmission has continued. Once the putative coronavirus, now named severe acute respiratory syndrome coronavirus 2 (SARS-CoV-2), had been identified in China in late 2019 [4], countries responded with different senses of urgency. When person-to-person transmission was confirmed, China immediately locked down the ca 57 million persons living in the Wuhan Province and then extended the lockdowns to other parts of China where transmission was identified in an attempt to attain and maintain zero transmission of SARS-CoV-2 [4]. Many other countries in Asia had begun identifying and stopping outbreaks of SARS-CoV-2 by mid-January with outbreak investigation and containment, and in some instances precision lockdowns where transmission was shown to be occurring such as nightclubs and gyms in Japan and churches in South Korea. In much of the rest of the world, this did not occur similarly at this stage as transmission increased and heathcare systems became overwhelmed with patients with ventilator demand and increasing deaths, as occurred early on in Italy and consecutively other countries in Europe. Many countries used general lockdowns, as in China, to slow transmission and decrease hospital burden and save lives. In addition to other outbreak control measures such as outbreak investigation and contact tracing, many countries in Europe and elsewhere also closed borders to international travel [5], or established strict border controls.

In this situation, several Asian countries that had previous experience with epidemics of respiratory disease, including influenza and SARS and to a certain extent Middle East respiratory syndrome (MERS), found that they could rely on national pandemic preparedness plans that took these epidemics into account. After these experiences, for example, they built physical infrastructure that took into account the need for a surge capacity, as well as organisational structures across government that facilitated a multisectoral and whole-of-government approach based on those structures that had been established and tested during the emergency response to previous epidemics [6]. In a few of these countries, national simulation exercises had been conducted and stockpiles of personal protective equipment and essential medicines had been secured for initial use over several months [6,7]. Some countries, such as New Zealand and Australia, began putting lockdowns in place in an attempt to obtain zero transmission following the China example [8].

In European countries, the preparedness focus had been mainly on an influenza pandemic, with exercises and pandemic planning for influenza alone and not for other respiratory infections such as those caused by coronaviruses [9]. Over the past 2 years, experiences have illustrated that national responses to COVID-19 are dependent on multiple interacting factors with a multi-sectoral whole-of-government approach, multi-level governance, apt and trusted leadership, swift coordination, well organised scientific advice, resilient health systems, and community participation in most successful responses [10].

At the same time, innovation in Europe, North America, and many other parts of the world, stimulated by massive upfront funding for research and development, rapidly led to development of new vaccines, antivirals, monoclonal antibody preparations and understanding the benefit of existing steroids—in particular dexamethasone [11]. Simultaneously, innovation in diagnostic testing led from initial PCR testing for nucleic acid detection to lateral flow antigen detection and self-testing [12]. Such innovations helped attain better pandemic control, and at the same time classical serological testing for antibody has played a role in understanding the SARS-CoV-2 epidemiology [13]. To ensure that these goods are more equitably distributed to where they are needed, the Access to COVID Tools Accelerator (ACT-A) was launched in April 2020 by the World Health Organization and partners as a facility to support research and development, and to help shape the market by offering a mechanism through which all countries and donor agencies could purchase COVID-19 vaccines, diagnostic tests and therapeutics [14]. Though it was not able to capture the massive upfront funding provided by high income countries directly to manufacturers in pre-purchase agreements, it has by early March 2022 distributed over 1.36 billion doses of vaccine [15], and over 114 million diagnostic tests to lower and middle income countries with donor funding, while at least one industrialised country announced that it had purchased a million doses of vaccine to meet an immediate demand [16].

There have also been other innovations such as digital contact tracing using phone apps that have had varied success depending on legal enablers and trust of populations [12], while the sharing of genetic sequence data on global platforms such as the Global Influenza Surveillance and Response System (GISAID), the International Nucleotide Sequence Database Collaboration (INSDC) and GenBank has permitted real-time understanding of variants and their impact. The World Health Organization Emergency Use Listing (EUL), an innovation after the Ebola outbreaks in West Africa, has been widely used to rapidly approve new COVID-19 vaccines, with regulators and developers working side by side rather than in sequence as is often the case for non-emergency regulation. Finally, many countries have focused on coordinated health service delivery and public health functions in communities such as testing, contact tracing, quarantine and treatment with attendant socioeconomic supports for individuals and families. In countries with successful responses, this was underpinned by high levels of trust in governments and political leaders by the general public [17]. In many countries, health workers themselves stepped in to provide innovative solutions to help reorganise hospitals and intensive care units, set up telemedicine services in primary healthcare, manage COVID-19 patients, provide palliative care in homes, and provide health messaging [18].

Throughout the pandemic response, scientific advice and the use of evidence has been needed to guide policy decisions. Many countries have relied on existing institutions to collect and translate emerging evidence into action such as the European Centre for Disease Prevention and Control (ECDC) and the European Medicines Agency (EMA), establishing temporary COVID-19 advisory groups, task forces, and panels of experts to inform government decision making, often at the level of the cabinet. Having scientific committees working together and reporting to the highest level of government, and making clear non-conflicting information available publicly, has helped countries make decisions based on evidence and gain the trust of the population [9].

Recently, governments have started shifting risk assessment and management to the population and attempted to control SARS-CoV-2 as an endemic infection, as is done for influenza and other respiratory diseases. In the UK and Germany, for example, free lateral flow kits were provided to empower individuals to do their own risk assessment, and vaccinations were widely promoted and accepted [19]. Endemicity of SARS-CoV-2, defined as the stable maintenance of the virus within a population [20], does not permit complacence because like influenza, there may be periodic resurgence of transmission as well as continued mutation and new variants emerging. The transition from pandemic to endemic will likely play out differently in different locations around the world, and each country might therefore have a different interpretation of what it means to live with endemic SARS-CoV-2 [20].

Governments that make the shift from pandemic response to endemic control must ideally have in place vaccination programmes, influenza-like illness (ILI) surveillance supported by genetic sequencing, public health capacity to investigate and contain outbreaks, and a focus on severe acute respiratory infection (SARI) among hospital admissions and deaths so that changes in strategy, albeit up to 2 or more weeks after a surge in transmission begins, can be rapidly put into place if seriousness of disease increases. Other surveillance activities might include environmental surveillance to identify and genetically sequence SARS-CoV-2 from sewage, syndromic surveillance to look for clusters of respiratory illness, and some type of participatory surveillance as has been recommended by a group of European surveillance experts [19,21,22].

A major factor of success depends on vaccines, how populations are protected against infection over time, and global vaccine equity [23]. ‘Herd immunity’ that would decrease transmission to very low levels was misunderstood before COVID-19 vaccines became available and before infections had been shown to not prevent reinfection. It is now known that ‘population immunity’ is a better term than herd immunity because though vaccines prevent serious illness in those infected post-vaccination, they do not prevent infection as do vaccines for measles, for example. The same is true for infection of naïve individuals – it does not provide lasting protection against reinfection. But the level of population immunity, as measured by serological surveys, helps predict outcomes as countries develop control programmes. High population immunity such as present in the UK, where over 98% of the population is thought to have antibody based on regular serological surveys, can be assumed to prevent against serious illness caused by known variants [24]. Additionally, ILI, SARI and other types of surveillance with genetic sequencing ensures that if future variants escape population immunity, vaccines can be adjusted as needed.

Learning from the COVID-19 pandemic must now be translated into action. In particular, top in the mind of all decisionmakers should be the need to strengthen preparedness by establishing strong national capacity in three interlocking areas within a health system: (i) strong public health capacity to detect and respond to emerging infections where and when they occur; (ii) resilient health systems that can accommodate both those infected during a pandemic as well as those requiring routine management and care; and (iii) an enabling environment for healthy lifestyles to ensure that populations are able to fend off serious illness when infected [25]. These three interlocking functions need to be properly integrated and complemented by a One Health, whole-of-government approach, with rapid coordination and well-organised scientific advice, and with sustained efforts to involve communities and vulnerable populations in the pandemic response. A One Health approach recognises that the health of people is connected to the health of animals and our shared environment and requires cooperation across the human, animal, and environmental systems [26].

To provide the best possible health outcomes when and if pandemics occur in the future, key stakeholders should work closely together nationally to reorient health systems so that they are resilient to shocks by integrating public health, patient management and health promotion from the central government all the way to the community level. At the same time, key stakeholders should assess what was not effective during their response to this pandemic by ensuring adequate evaluation mechanisms.

At the global level, a package of reforms is needed to raise additional financing for preparedness and for surge funding in case of a pandemic, and to establish mechanisms to ensure that pandemic threats are raised to the highest leadership level [27]. While regional and global mechanisms are required to ensure free and responsible sharing of data and scientific advice and equitable access to the goods required for national preparedness, emphasis should also be on establishing capacity at the country level.

The lessons of the pandemic are clear. Both the global and national public health communities must remain in constant dialogue with political decision makers to be sure they are heard and remembered by political leaders and decision makers.
